# Age-related neurochemical changes in the rhesus macaque inferior colliculus

**DOI:** 10.3389/fnagi.2014.00073

**Published:** 2014-04-21

**Authors:** James R. Engle, Daniel T. Gray, Heather Turner, Julia B. Udell, Gregg H. Recanzone

**Affiliations:** ^1^Evelyn F. McKnight Brain Institute, University of Arizona at TucsonTucson, AZ, USA; ^2^Center for Neuroscience, University of California at DavisDavis, CA, USA; ^3^Department of Psychology, University of California at DavisDavis, CA, USA; ^4^Department of Neurobiology, Physiology and Behavior, University of California at DavisDavis, CA USA

**Keywords:** inferior colliculus, parvalbumin, NADPHd, hearing deficits, aging

## Abstract

Age-related hearing loss (ARHL) is marked by audiometric hearing deficits that propagate along the auditory pathway. Neurochemical changes as a function of aging have also been identified in neurons along the auditory pathway in both rodents and carnivores, however, very little is known about how these neurochemicals change in the non-human primate. To examine how these compensatory neurochemical changes relate to normal aging and audiometric sensitivity along the auditory pathway, we collected auditory brainstem responses (ABRs) and brain specimens from seven rhesus monkeys spanning in age from 15 to 35 years old, and examined the relationship between click evoked ABR thresholds and the ABR evoked pure tone average (PTA) and changes in the number of parvalbumin and NADPH-diaphorase positive cells in the auditory midbrain. We found that the number of parvalbumin positive cells in the central nucleus and the surrounding cortex regions of the inferior colliculus were strongly correlated with advancing age and ABR PTA. We also found that the numbers of NADPHd positive cells in these same regions were not associated with normal aging or changes in the ABR thresholds. These findings suggest that the auditory midbrain undergoes an up-regulation of parvalbumin expressing neurons with aging that is related to changes in the processing of frequencies across the audiometric range.

## Introduction

Age-related hearing loss (ARHL) is characterized by spectral, temporal and spatial processing deficits (Caspary et al., 1995, 2008; Frisina and Frisina, 1997; Snell, 1997; Abel et al., 2000a,b; Juarez-Salinas et al., 2010). These processing deficits have been linked to a degradation of the peripheral elements of the cochlea (Engle et al., 2013), and/or a decline of central inhibitory mechanisms that lead to changes in neuronal excitability within the auditory system (Caspary et al., 1995, 1999, 2008). Several elegant neuroanatomical studies in rodents have characterized the function of aging on the auditory system by examining the neurochemical changes associated with the calcium binding proteins calbindin (CB) and parvalbumin (PV), the nitric oxide synthase Nicotinamide Adenine Dinucleotide Hydrogen Phosphate Diaphorase (NADPHd), and glutamic acid decarboxylase (GAD). Changes in the expression of these molecules have been described in the cochlear nucleus, superior olivary complex, inferior colliculus, medial geniculate body, and the auditory cortex (O'Neill et al., 1997; Zettel et al., 1997; Ouda et al., 2003; Caspary et al., 2005, 2006; Ouda et al., 2008; de Villiers-Sidani et al., 2010). The role of these neurochemical changes throughout the lifespan in rodents remains unclear, and there is very little knowledge of how specific age-related neurochemical changes relate to ARHL in the primate. Given the current interest in studying the macaque auditory system as a model for human auditory perception (e.g., Kaas and Hackett, 2000; Rauschecker and Tian, 2000) and the more recent interest in the effects of aging on macaque auditory processing (e.g., Juarez-Salinas et al., 2010; Recanzone et al., 2011; Engle and Recanzone, 2012; Engle et al., 2013) it is important to understand the similarities and differences between these two animal models. Previous studies from this laboratory quantifying the numbers of neurons expressing PV and NADPHd in the macaque cochlear nucleus (Gray et al., 2014b), superior olivary complex (Gray et al., 2014a) and auditory thalamus (Gray et al., 2013) suggest that, while age-related changes are noted, they are not identical to those found in rodents. The aim of the present study is to characterize the neurochemical changes in the inferior colliculus, the obligatory relay nucleus of the ascending auditory central nervous system, of the aged macaque monkey to gain a better understanding of the anatomical correlates that accompany aging in the primate auditory system.

One approach to understand age-related changes in the auditory system has been to consider how neurochemical markers common to the auditory system change with natural aging. The central auditory system of the macaque monkey has been segregated into chemically defined parallel processing streams (Jones, 2003). As reviewed by Jones, the “direct” auditory pathway originates in the ventral nucleus of the cochlear nucleus and ultimately terminates in the core of the auditory cortex. This pathway is characterized by its dense immunoreactivity for PV in both cells and the neuropil and ascends through the central nucleus of the inferior colliculus (ICC). In contrast, the tegmental “indirect” pathway that ascends to the core and belt fields of auditory cortex is characterized by CB, which is primarily found in the surrounding cortex of the inferior colliculus (ICX). The parcelation of auditory structures based upon similar organizational characteristics was recently described in rodents and carnivores with cytochrome oxidase and NADPHd (Loftus et al., 2008). In these parallel pathways, the relative density of neurons that express either PV and/or NADPHd have been shown to change with age in rodents and non-human primates (Zettel et al., 1997; Reuss et al., 2000; Ouda et al., 2003, 2008; Sanchez-Zuriaga et al., 2007; Huh et al., 2008; Gray et al., 2013, 2014a,b), and are thought to play a compensatory role in response to decreased output from the cochlea and/or to changes in neuronal excitability (Gray et al., 2014b).

One useful approach to understand the compensatory nature of these neuronal populations in the aging auditory system is to consider that both PV and NADPHd are dependent upon or regulate calcium, which plays an important role in neurotransmission. Age-related studies in rodents have found a decline of GABAergic and glycinergic inhibitory mechanisms throughout the central auditory system (Caspary et al., 1995, 2008), thus leading to an increase in neuronal excitability. Neurons that express PV and NADPHd may compensate for changes in excitability by buffering calcium, or by signaling through the production of nitric oxide. It is unclear if these neurochemicals of interest colocalize with GABAergic and glycinergic inhibitory neurons in the auditory brainstems. Distinct classes of GABAergic interneurons, however, have been identified by their neurochemical signature for the calcium binding proteins PV, CB and calretinin in the cerebral cortex and hippocampus (Carder et al., 1996). Fredich et al. (2009) reported that these calcium binding proteins colocalized with GABA and glycine in a region specific manner in the auditory brainstem of Wistar rats. Importantly for the present study, Fredich et al. found that all GABAergic cells in the ICC co-localized with PV, and that only a small percentage of PV cells lacked this profile, although similar studies have not been reported in primates. Age-related changes in the number of these specific calcium binding proteins have been found throughout the central auditory system (O'Neill et al., 1997; Zettel et al., 1997; Ouda et al., 2008). Unfortunately, age-related changes of these neurochemical markers are not consistent throughout the auditory system, and appear to depend on the species as well as the particular auditory nucleus and area. For example, Zettel et al. (1997) found that neurons staining positively for CB and calretinin increased with age in regions outside of the central nucleus of the inferior colliculus of CBA and C57 mice. Ouda et al. (2008) found an age-related change in the number of PV positive cells that was dependent upon the auditory area (i.e., the inferior colliculus vs. auditory cortex) and the type of rat (i.e., Long Evan vs. Fischer 344). More specifically, they found that the number of PV positive cells increased in the inferior colliculus of aged Long-Evan rats, and decreased in the auditory cortex of aged Fischer 344 rats. de Villiers-Sidani et al. (2010) showed an age-related decrease in PV positive neurons in auditory cortex that correlated with cortical detuning of auditory cortical receptive fields. Interestingly, de Villiers-Sidani et al. (2010) also showed that both anatomical and physiological correlates could be significantly mitigated by auditory training. Therefore, an age-related change in the relative density of neurons that express these calcium binding proteins may serve to buffer and preserve auditory processing in the face of increased neuronal excitability.

NADPHd has been identified as a nitric oxide synthase (Bredt et al., 1991; Dawson et al., 1991a), and NADPHd positive cells are thought to regulate and be resistant to glutamate excitotoxity (Dawson et al., 1991b). NADPHd has also been shown to co-localize with distinct calcium binding proteins (Yan et al., 1996), and shows age-related changes throughout the auditory system. Age-related changes in the number of the NADPHd positive cells have been found in the cochlear nucleus of macaques (Gray et al., 2014b), the superior olivary complex of macaques (Gray et al., 2013) and chinchillas (Reuss et al., 2000), as well as the inferior colliculus and auditory cortex of rats (Ouda et al., 2003; Sanchez-Zuriaga et al., 2007; Huh et al., 2008).

Previous studies in rodents and monkeys have shown that differentiation and temporary sensory deprivation can result in quantifiable changes to the population of PV cells in both the auditory and visual systems (Carder et al., 1996; Caicedo et al., 1997). These studies suggest that specific neuronal populations (i.e., GABAergic interneurons) can be directly modulated by the functionality of the peripheral sensory organ and how well it is connected to its downstream target(s). ARHL is characterized by changes in auditory sensitivity and in many cases by histopathological changes of the cochlea (e.g., Schuknecht, 1964; Schuknecht and Gacek, 1993; Engle and Recanzone, 2012). It is therefore conceivable that age-related changes in cochlear function and histopathologies provoke up-regulation or down-regulation of neuronal populations in downstream auditory areas to compensate for changes in decreased output from the cochlea and/or for changes in neuronal excitability. Compensatory changes in neuronal populations have been found as early as the cochlear nucleus of aged rhesus macaques. Gray et al. (2014b) found specific correlations between the densities of different neuronal populations, age, cochlear function and cochlear histopathology to support this hypothesis. For example, Gray et al. found that the density of PV positive neurons was inversely related to ABR amplitudes. The proposed compensatory effects would presumably progress from stage to stage, resulting in changes in the fidelity of the auditory information that is being processed. Therefore, changes in specific neuronal populations in relation to changes in auditory sensitivity may be indicative of a possible compensatory mechanism to maintain the fidelity of auditory information passing along the auditory system.

The inferior colliculus is an ideal auditory structure to study as it is the obligatory relay nucleus between multiple brainstem processing areas and the auditory thalamus and cerebral cortex (Kaas and Hackett, 2000). In addition, a previous study from this laboratory showed that the medial superior olive (MSO) of the superior olivary complex (SOC) showed age-related increases in the number of both PV and NADPHd reactive neurons, and a correlation of the overall number of the positively stained neurons across the SOC and ABR thresholds measured in the same monkeys (Gray et al., 2013, 2014b). As the SOC is one of the major inputs to the inferior colliculus, the question is raised whether similar changes occur at this higher level auditory area as well. Here, we hypothesized that the prolonged age-related decrease in auditory sensitivity leads to compensatory changes expressed by the number of neurons expressing these neurochemical markers in the inferior colliculus. The ABR was used to assess auditory sensitivity, and then anatomical specimens were obtained to help understand how aging and ARHL engages these compensatory processes in the auditory system of the rhesus monkey. Changes in auditory sensitivity across the audiometric range was assessed by generating an ABR pure tone average (PTA). This metric of auditory sensitivity and chronological age was then correlated with changes in the numbers of neurons with PV immunoreactivity and NADPHd histochemical reactivity in the inferior colliculus of the rhesus monkey to determine whether a change in audiometric sensitivity leads to structural compensatory signatures.

## Materials and methods

The distributions of the calcium binding protein PV and NADPHd positive neurons in the inferior colliculus were examined in 7 adult macaque monkeys (*Macaca mulatta*) using stereological sampling techniques. The age of the monkeys ranged from 15 to 35 years of age. These animals are a subset of those previously reported (Engle and Recanzone, 2012; Gray et al., 2013, 2014a,b) and the overlapping methods are summarized here. The 20 and 27 year old monkeys had extensive auditory training by participating in several unrelated auditory experiments. All animals were housed under standard, Institutional Animal Care and Use Committee (IACUC) approved conditions. Three of the seven animals were euthanized for unrelated medical reasons, three were euthanized as part of terminal procedures for unrelated experiments, and the remaining was euthanized specifically for this project. All animals were maintained on *ad libitum* food and free access to water. Criteria to be included in the study were: (1) No known history of ototoxic drug exposure; (2) No known history of loud noise exposure or traumatic injury to the ear; (3) ABRs having been recorded within 6 months of euthanasia; and (4) No outer ear occlusions or signs of otitis media during otoscopic examination. All experimental procedures conformed to the National Institutes of Health guidelines for animal use, and were approved by the University of California, Davis IACUC.

### Auditory brainstem response procedure

All recordings were obtained in a quiet electrically shielded room or in a double-walled sound booth. Each animal was lightly anesthetized with ketamine (10 mg/kg, IM) and medetomidine (0.3 ml/10 kg, IM) to maintain chemical restraint during these non-noxious procedures. The depth of anesthetic was monitored by heart rate, blood oxygenation level, respiratory rate, and lack of muscular movement. Each animal was given additional injections of ketamine to maintain chemical restraint throughout the procedure if necessary, which ranged from 1 to 3 h. The animal was placed in the prone position with their head slightly elevated. The ear canals were inspected and cleaned of debris if necessary to determine the condition of the tympanic membrane. Electrodes (0.22 gauge stainless steel sterile wires) were placed subcutaneously behind each ear, on the forehead, and on the back of the neck (Allen and Starr, 1978; Torre and Fowler, 2000; Torre et al., 2004; Fowler et al., 2010). ABR recordings were obtained using an Intelligent Hearing System (IHS; Smart EP Win USB, Version 3.97, Miami, FL) interfaced with a laptop computer to control stimulus delivery and data acquisition. ABRs were amplified at 100 K times and filtered at 100–1500 Hz. Soft earphones (etymotic ER3A) were placed in both the left and right ear. Click and pure tone stimuli at 0.5, 1, 2, 4, 8, 12, and 16 kHz were binaurally presented at a rate of 50 stim/s at a minimum of 1000 repetitions to obtain a reliable average waveform. Stimulus amplitude started at 80 dB SPL and decreased in 10 dB SPL increments until the ABR response was no longer visually identifiable, and then increased by 5 dB SPL. Threshold was defined as the midway point between when the response was visible and when it was not, and therefore is within approximately 2.5 dB SPL. ABR threshold, peak and latency measurements were manually scored by two independent raters (95% inter-rater reliability) who were blind to the age and identity of the monkeys. ABR thresholds and latencies were usually symmetric between the ears and were not systematically different across tested frequencies in any animal tested. Therefore, threshold was reported from the ear with the lowest threshold. ABR thresholds across frequencies were averaged across all seven frequencies, and are expressed as a seven frequency PTA.

### Histological processing

**Figure 1:**
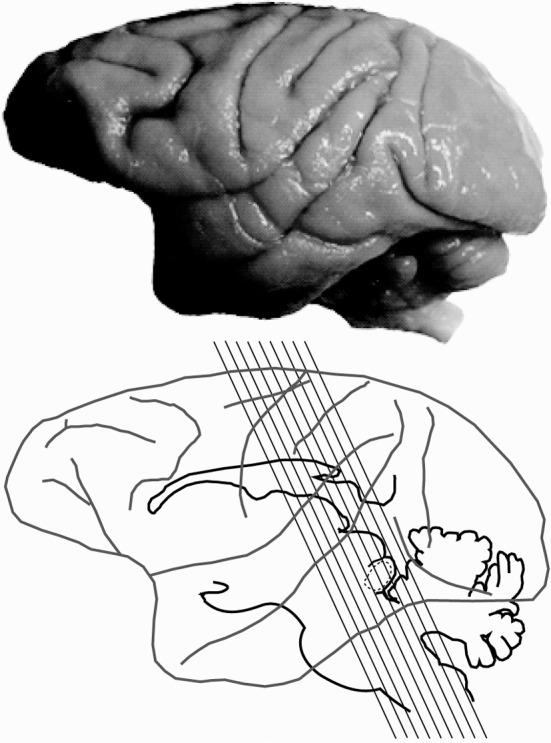
**Planes of section of the inferior colliculus**. Lateral view of the macaque brain illustrating the plane of section through the inferior colliculus (IC) for 6 of the 7 specimens.

Histological processing followed those detailed previously (Gray et al., 2013, 2014b) and are briefly summarized here. Each animal was anesthetized with ketamine (30 mg/kg, IM), and euthanized with a lethal dose of sodium pentobarbital (60 mg/kg, IV). Each animal was then transcardially perfused with saline, followed by two fixatives. Fix 1 contained a mixture of 4% paraformaldehyde and 0.1% glutaraldehyde in 0.1 M phosphate buffer (pH 7.4), and fix 2 contained a mixture of 4% paraformaldehyde and 10% sucrose. The brains were then removed and post-fixed at 4°C in a mixture of 4% paraformaldehyde and 30% sucrose for cryoprotection. Each brain was then prepared for light microscopy, and sectioned at 25–50 μm on a freezing microtome, washed and stored in 0.1 M phosphate buffer before tissue processing (Hackett et al., 2001; de la Mothe et al., 2006a,b; Padberg et al., 2009). Six of seven cases were cut perpendicular to the lateral sulcus (Figure 1), and in one case we extracted the midbrain and thalamus from the neocortex and cut 50 μm sections in the transverse plane to reveal rostral to caudal progressions of the inferior colliculus (see Table 1 for details). Serial sections were cut through the entire brain in an alternating fashion of 6–12 to capture the relative distribution of neurochemicals of interest per brain. The method yielded 10–12 sections through the IC per series. Two of these series were then selected at random and processed individually for PV immunohistochemistry (Parvalbumin, Sigma-Aldrich cat no PV3088, mouse monoclonal, 1:4000) and NADPHd histochemistry, while the remaining sections were processed and used for another study.

**Table 1:** **Demographics, auditory brainstem response and anatomical methods used in monkeys**.

**Age(mo)**	**Gender**	**Auditory trained**	**ABR Click**	**ABR PTA**	**Plane of section**	**Thickness (μm)**
184	Male	No	45	39.29	Sagittal	40
185	Male	No	35	53.57	Oblique	30
243	Female	Yes	30	50.51	Oblique	50
255	Female	No	45	55.00	Oblique	50
267	Female	No	40	58.57	Oblique	50
324	Male	Yes	30	51.42	Oblique	40
427	Female	No	70	71.43	Oblique	25

Immunohistochemical reactions followed the ABC method (Vectastain ABC Kit, Vector Labs, Burlingame, CA) and were visualized with 3,3′ diaminobenzidine (DAB) or the Vector SG kit (Vector labs, CA). NADPHd histochemistry was performed by following a modified histochemical procedure to reveal NADPHd activity (Scherer-Singler et al., 1983). Briefly, free floating sections were incubated in the dark for 1 h at room temperature in a solution containing 1 mg/ml of nitroblue tetrazolium (Sigma Aldrich, N-6876), 0.5 mg/ml of β-nicotinamide adenine dinucleotide phosphate (NADPH; Sigma-Aldrich, N-1630), 0.1% Triton X-100 dissolved in 0.1 M phosphate buffer. Sections were then rinsed in 0.1 M phosphate buffer, mounted on subbed glass slides, air dried and then cover slipped with DPX.

### Antibody characterization

The isotype specificity of the anti-PV antibody was determined using the Sigma immunoType™ Kit (Product Code ISO-1), and by a double diffusion immunoassay using Mouse Monoclonal Antibody Isotyping Reagents (Product Code ISO-2). Negative controls in which either the primary antibody (PV immunohistochemistry) or nitroblue tetrazolium (NADPHd histochemistry) were omitted revealed no positive signal. All antibodies, immunogen specificity, and chemicals used in these procedures are summarized in Table 2.

**Table 2:** **Antibody and chemical list used for immunohistochemical and histochemical reactions**.

**Method**	**Antibody/Chemical**	**Immunogen structure**	**Manufacture information**
Parvalbumin immunohistochemistry	Monoclonal anti-parvalbumin clone PARV-19	PARV-19 hybridoma	Sigma-Aldrich, P-3088; monoclonal; raised in mouse
Parvalbumin immunohistochemistry	Biotinylated anti-mouse IgG	Mouse IgG	Vector labs, BA-2000; made in horse
Parvalbumin immunohistochemistry	Normal horse serum	N/A	Vector labs, S-2000
Parvalbumin immunohistochemistry	SG- substrate kit	N/A	Vector-labs, SK-4700
NADPH-diaphorase histochemistry	Nitroblue tetrazolium	N/A	Sigma-Aldrich, N-6876
NADPH-diaphorase histochemistry	β-nicotinamide adenine dinucleotide phosphate (NADPH)	N/A	Sigma-Aldrich, N-1630

### Reconstruction and data analysis

Standard light microscopy techniques were used in conjunction with techniques based upon stereological procedures to estimate age-related changes in the number of PV and NADPHd positive neurons. Data analysis was performed in two stages before being combined into a comprehensive reconstruction of the IC. First, stained sections from the right IC were analyzed by an investigator that was blind to the ages of the monkeys with a Nikon D8000 microscope that was equipped with an MD3-plot x/y stage encoding system (Accustage Inc, Shoreview, MN) that was connected to a personal computer. Tissue outlines, blood vessels and tissue artifacts were drawn at a low 4x magnification for PV and NADPHd mounted sections for each case. A grid with 400 μm spacing between points was then digitally overlaid, and then the section was printed for recording purposes. The Cavalieri probe integrated with an optical disector (100 μm^2^ unbiased counting frame) was used at 20x (Nikon Plan Fluor 20x/0.50 N.A. DIC M/N2, 8/0.17, WD 2.1 mm) magnification at each point along the grid that intersected the IC to estimate the volume (mm^3^) of the IC and to perform live counts on approximately 1/8 of the tissue for each section. At each point, the tissue was examined by focusing through the tissue from top to bottom without a guard zone. A cell was counted if the neuronal cell body was stained and within the unbiased counting frame without touching the exclusionary boundary of the frame, and the number of cells was recorded at each point. This sampling strategy was selected to derive an estimated average cell number per mm^3^/section to reduce to experimental and experimenter error that may have been introduced between cases.

Next, we confirmed our ability to reliably differentiate positively stained cells from the neuropil and background staining (Gray et al., 2013), and to rule out an overestimation bias created by differences in tissue thickness across cases (Mounton, 2002). To accomplish this, we randomly resampled the tissue and counted neuronal cells offline according to the method above. Images were then taken at several planes (3–5 images per point) of each section to span the entire height of the optical disector with a guard zone of 5 microns that equaled ¼ of an averaged cell height (Mounton, 2002; Gray et al., 2013). The images were then imported into Adobe Photoshop, grayscaled, thresholded, and binarized so that values of 1 (black) represented the lowest intensity value, which corresponded to a stained cell, and the rest of the image was given a value of 0 (white). Cell counts of stained neurons were then performed with this method by a second counter. We found that our cell counts between both methods were in agreement over 95% of the counts (See Figure 4 of Gray et al., 2013). The result of this analysis indicates that the counters reliably differentiated positively labeled cells from background and neuropil staining, and overestimations of neuron counts were unlikely.

In the second stage, region of interest (ROI) anatomical borders were identified by gross chemoarchitecture and drawn using a projection microscope. ROI images were then digitized, rescaled, overlaid to match the tissue printouts, which were then manually registered with MD-plot tracings by aligning tissue outlines, blood vessels, and tissue artifacts in Adobe Illustrator to reconstruct the IC. The volume of each section through the IC was then estimated using the Cavalieri principle (Howard and Reed, 2005) as:

$$\text{V}(\text{ref})=\text{A}/\text{P}*\Sigma_{\text{p}}*\text{T}$$

Where A/P is the area between adjacent points on the grid, ΣP is the sum of the points in each area per section, and T is the tissue thickness. The total number of positive neurons per section was estimated by:

Ns=(ΣQ/N×Vdis)∗V(ref)

Where ΣQ is the sum of cells counted, N is the number of observation points, and Vdis is the volume of the optical dissector. From these values we were able to estimate the number of stained neurons within a given structure in each animal. It should be noted that “number” is therefore referring to the number of positively stained neurons in a given volume, and is independent of the degree of staining of individual neurons.

Statistical analysis was performed in two steps by using SPSS, version 20 (Chicago, IL). We collapsed the data into an average per case, calculated the Pearson product moment correlation, and then used multiple regression analysis to tease apart the effect of age and ABR threshold on the number of PV positive and NADPHd positive cells in the core and the shell of the IC. We used a criterion of *p* < 0.05 to enter and a *p* > 0.10 to remove predictors from the model. Values that fell between 0.05 and 0.10 stayed in the analysis as a trend. We used age, ABR waveform IV threshold, waveform IV latency at threshold and waveform IV amplitude at threshold as predictors of the number of neurons in the core, the shell and the ratio between the core and shell of the IC.

## Results

### Auditory brainstem response threshold increases with advanced age

**Figure 2:**
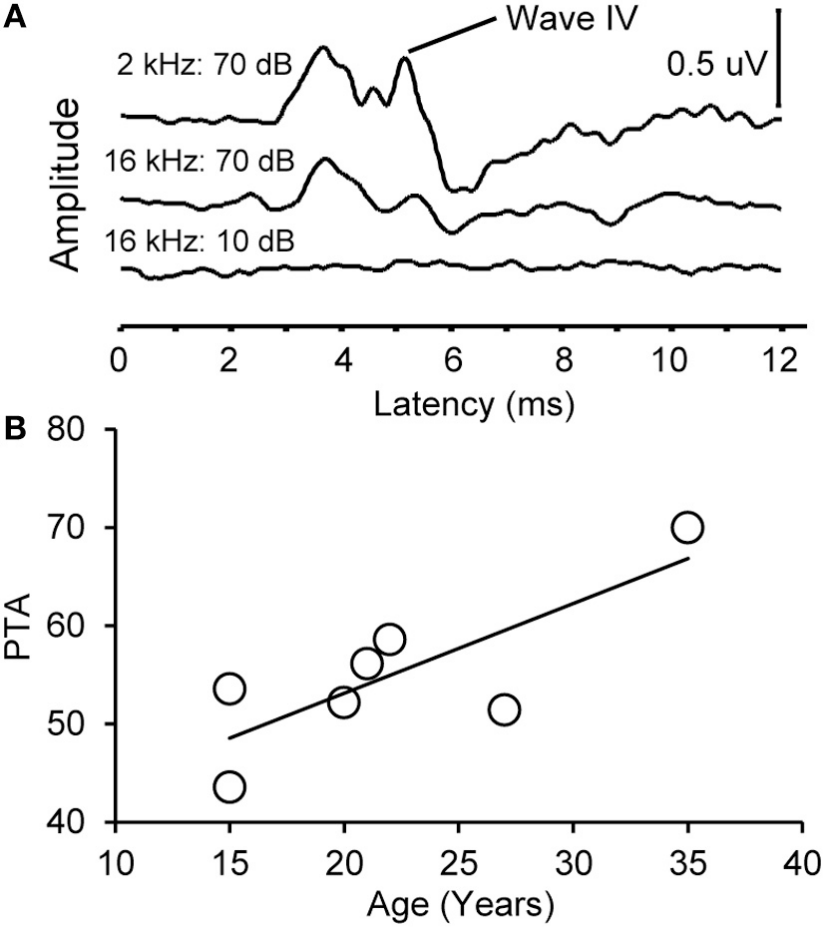
**Example auditory brainstem responses and pure tone average audiogram**. **(A)** Representative ABR waveform from a 15 year old monkey at 2 and 16 kHz at 70 dB SPL (top) and 10 dB SPL (bottom). Wave IV is highlighted. Note that the ABR response at all levels was present at the higher intensity stimulus and absent in the lowest intensity stimulus. **(B)** Pure tone average (PTA) as a function of age. PTA was the average ABR wave IV threshold across tone frequencies tested (0.5–16 kHz, see Methods). There was a statistically significant increase in PTA as a function of age.

We collected ABR recordings from all seven monkeys (see Table 1). Rhesus macaques age at an approximate rate of 3:1 compared to humans (see Davis and Leathers, 1985), so the animals in this study were approximately 45–107 human years of age. We choose to examine the threshold of wave IV because the IC presumably contributes most to the activity of the ABR wave IV signal (Allen and Starr, 1978; Laughlin et al., 1999). We also considered using wave I to indicate general hearing ability for these animals, but found that the amplitude and latency of this peak was much more variable and smaller than wave II and IV due to the fast stimulus rate, which made it more difficult to accurately score by the two independent observers, particularly for the older animals. For these two reasons we restricted our analysis to wave IV of the ABR response. These results are illustrated in Figure 2. In the first layer of this analysis, we examined the Pearson product moment correlations to identify the relationship between age and the ABR threshold data (see Table 3). In this sample, we found that higher ABR click thresholds were associated with greater wave IV amplitudes (*r* = 0.86, *p* < 0.05), but not faster wave IV latencies (*r* = −0.36, *p* > 0.05). We found that ABR click thresholds had a non-significant trend to increase with advancing age (*r* = 0.62, *p* = 0.13). ABRs evoked by clicks are somewhat restricted across the audiometric range of monkeys, so we summarized the ABR response to tones by creating a seven frequency PTA. In this case we found that PTA was significantly correlated with age (*r* = 0.80, *p* < 0.05; Figure 2B). This result suggests that hearing thresholds systematically increased across the audiometric range with aging in these monkeys.

**Table 3:** **Correlation table between age, ABR data and anatomical counts of PV and NADPHd positive neurons in the inferior colliculus of monkeys**.

	**Age**	**Click thres**	**PTA**	**Parv ICC**	**Parv ICX**	**NADPHd ICC**	**NADPHd ICX**	**Inf. olive**
Age	1.00							
Click thres	0.62	1.00						
PTA	0.81^*^	0.82^*^	1.00					
Parv ICC	0.95^**^	0.61	0.78^*^	1.00				
Parv ICX	0.92^**^	0.42	0.71	0.89^**^	1.00			
NADPHd ICC	0.08	0.40	0.30	−0.15	−0.19	1.00		
NADPHd ICX	−0.31	−0.07	−0.34	−0.11	−0.49	−0.16	1.00	
Inf. olive	0.14	−0.51	−0.23	0.06	0.34	−0.35	−0.48	1.00

* *p < 0.05*,

** *p < 0.01*.

### Organization of the IC

**Figure 3:**
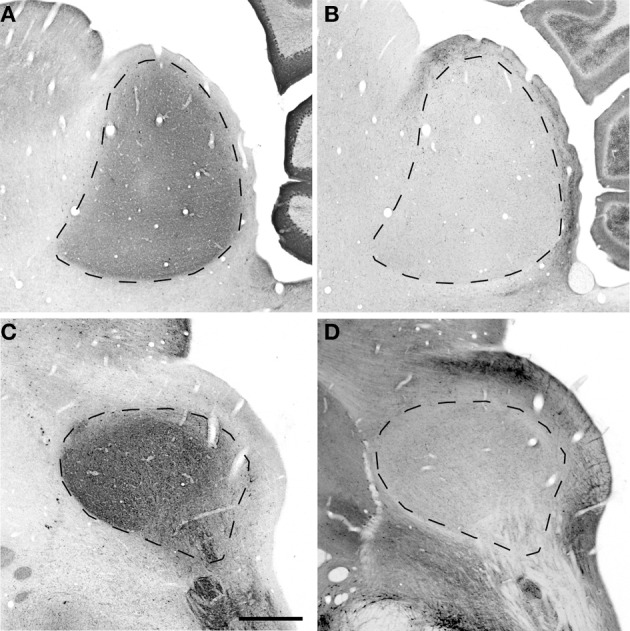
**Sagittal and oblique sections through the rhesus monkey inferior colliculus**. Example sections cut in the sagittal (15.3 years old; panels **A**,**B**) and oblique (15.4 years old; panels **C**,**D**). **(A,C)** Show PV immunostained sections where there is a dense staining of the core central nucleus of the IC. **(B,D)** Show NADPHd stained sections where there is dense staining of the ICX that surrounds the core of the IC. Dashed lines demarcate the border between ICX and ICC **(B,D)** and the external border of the ICX **(A,C)**. Scale bar = 1 mm.

To understand how aging and hearing loss impacts populations of neurons in the IC of the rhesus macaque monkey, we defined the IC anatomically into a central area that included the central nucleus (ICC), and a surrounding cortex (ICX) area that included the dorsal cortex (DC) and the lateral cortex (LC) conforming to the terminology used in physiological studies of the IC in macaque monkeys (Groh et al., 2003; Maier and Groh, 2010). The IC material in our hands was structurally organized in a manner consistent with what has been reported in the mouse, rat, cat, macaque and human (for review, see Oliver, 2005). The IC was located in the midbrain dorsal to the cuneiform nucleus and the nucleus of the lateral lemniscus, laterally to the periaquaductual gray, ventral and caudal to the superior colliculus and caudal to the central tegmental field. One characteristic of the IC in rodents, primates and humans is that chemoarchitecture can be used to parcellate the different subdivisions of the IC (Lohmann and Friauf, 1996; Jones, 2003; Parvizi and Damasio, 2003; Loftus et al., 2008). Figure 3 illustrates the ICC, DC and LC in oblique and sagittal sections stained for PV and NADPHd. Qualitative examination of these stained sections reveal that the differential staining pattern of PV positive and NADPHd positive cells and neuropil defines the subdivisions of the IC (Table 4).

**Table 4:** **Relative density of staining in the inferior colliculus of monkeys**.

**IC region**	**PV+ cells**	**NADPHd+ cells**	**PV+ neuropil**	**NADPHd+ neuropil**
**ICC**				
Central nucleus	+++	++	+++	
**ICX**				
Dorsal cortex	+	+++	+	+++
Lateral cortex	++	++	+	++

*^+^Denotes relative density of stained neurons*.

### Distribution of the calcium binding protein parvalbumin and NADPHD

The distribution of the immunostained PV positive cells and neuropil varied across the ICC and ICX of the IC (Figure 3). We found that the neuropil in the ICC was more densely stained for PV than the ICX and surrounding midbrain structures. This is quite apparent in Figures 3A,C. There was a considerable amount of overlap in the dorsal caudomedial junction between the ICC and the ICX, but this overlap was reduced in the remaining areas of the ICX. Interestingly, PV positive cells were not restricted to the densely stained neuropil of the ICC as we also found PV positive cells in the ICX. Quantification of these observations revealed that the ICC had significantly more PV positive cells than in the ICX (paired *t*-test, *p* < 0.001).

The distribution of histochemically stained NADPHd positive cells and neuropil varied in a complimentary fashion to what was found for PV in the IC. NADPHd staining of the neuropil was absent in the ICC and was restricted to the ICX. The most densely stained NADPHd positive neuropil was found in the caudal ICX, which wrapped dorsoventrally along the superficial caudal surface of the IC. However, this pattern did not extend all the way around the ICC. This is best observed in Figure 3B. We found patchy staining in the rostral lateral ICX, which suggests that the ICX of monkeys may be divided into multiple lamina as reported in rodents and carnivores (Loftus et al., 2008). The distribution of NADPHd positive cells followed a similar trend to the neuropil in the ICX of the IC.

**Figure 4:**
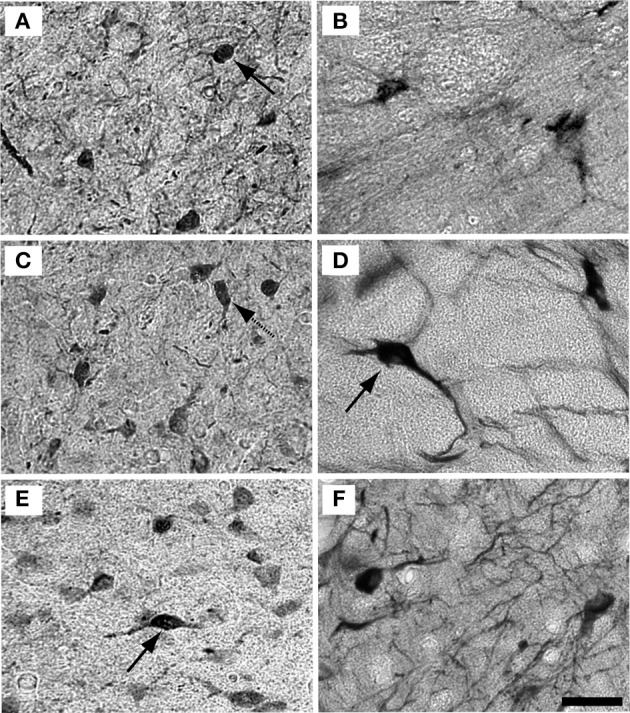
**Example high magnification images of changes in ICC staining with age**. Gray scaled images of PV+ (left) and NADPHd+ (right) cells in the core of the IC of the 15 **(A,B)**, 22 **(C,D)**, and 35 **(E,F)** year old monkeys. The number of PV+ cells increased with age (compare **A–C–E**) but not for NADPHd (compare **B–D–F**). Presumptive disc cells are labeled with solid arrows. Presumptive stellate cells are labeled with dashed arrows. Scale bar = 50 μm.

**Figure 5:**
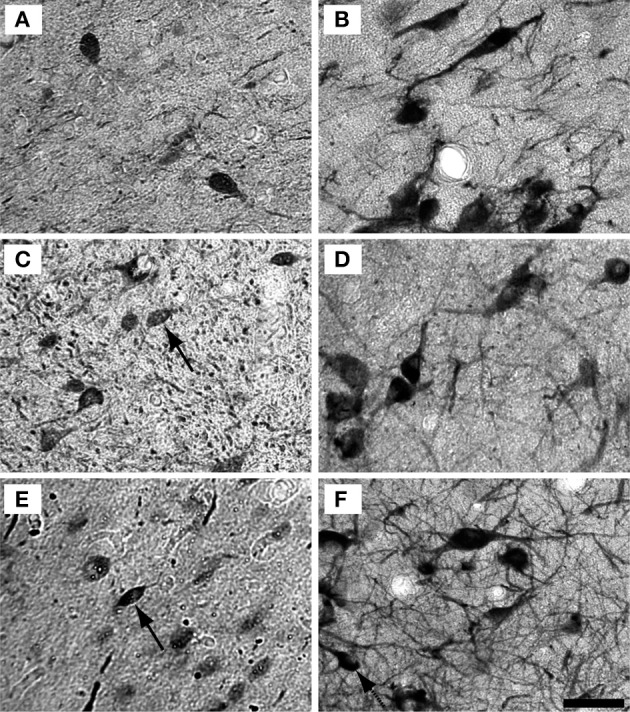
**Example high magnification images of changes in ICX staining with age**. Gray scaled images of PV+ (Left) and NADPHd+ (right) cells in the shell of the IC of the 15 **(A,B)**, 22 **(C,D)**, and 35 **(E,F)** year old monkeys. The number of PV+ cells appeared to increase with age, but not as much as in the ICC. As with the ICC, there did not appear to be any difference in NADPHd staining with age. Conventions as in Figure 4. Scale bar = 50 μm.

Examples of individual PV positive and NADPHd positive neurons in the central nucleus are shown in Figure 4. PV positive neurons found in the ICC were primarily presumptive disc and stellate cells, and NADPHd positive neurons were predominantly presumptive disc and stellate cells with sparse, more darkly stained multipolar neurons. In the representative 15 year old monkey (Figure 4A) PV positive cells were found in the ICC at a relatively low number, as were NADPHd positive (Figure 4B) cells. The number of cells staining positively for PV and NADPHd increased in the 22 year old monkey (Figures 4C,D) and the 35 year old monkey (Figures 4E,F), although more so for the PV positive cells. Examples of individual neurons in the dorsal ICX of the IC are shown in the same animals in Figure 5. In this region, neurons predominantly appeared as large or small multipolar neurons. In contrast to the ICC areas, there did not appear to a great difference in the number of stained neurons in the ICX as a function of age in these three examples.

Next, we quantified these initial impressions by estimating the number of PV positive cells and NADPHd positive cells per section of stained tissue through the IC (see Methods) to determine if the IC compensates for aging and/or hearing threshold. It should be noted that “number” refers to the number of stained neurons within a volume of tissue, and not the relative amount of staining within individual neurons.

### Age-related increase in the number of parvalbumin positive cells

**Figure 6:**
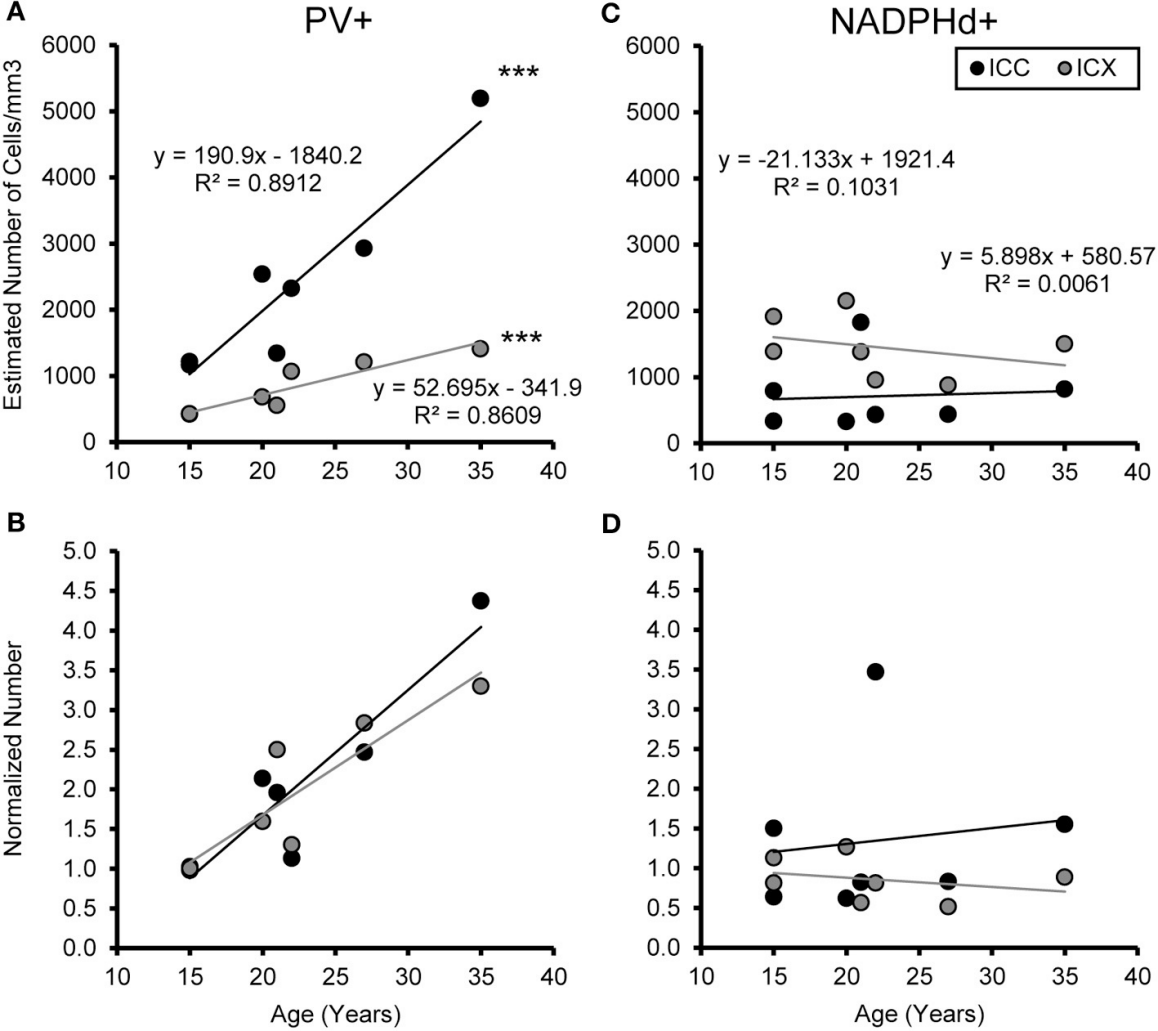
**Numbers of PV+ cells, but not NADPHd+ cells, increase with age in the ICC and ICX**. The estimated number of PV **(A)** and NADPHd **(C)** cells in the core (black) and shell (gray) of the IC as a function of age. For both the core and shell there was a significant correlation between the numbers of PV+ stained cells and age, but this was not true for the number of NADPHd+ stained cells. Normalizing the number to the average of the two youngest monkeys showed that the increases in the number of PV+ stained cells as a function of age was equivalent between the ICC and ICX **(B)**, but again there were no trends for the number of NADPHd+ stained cells **(D)**. ^***^p < 0.001.

**Figure 7:**
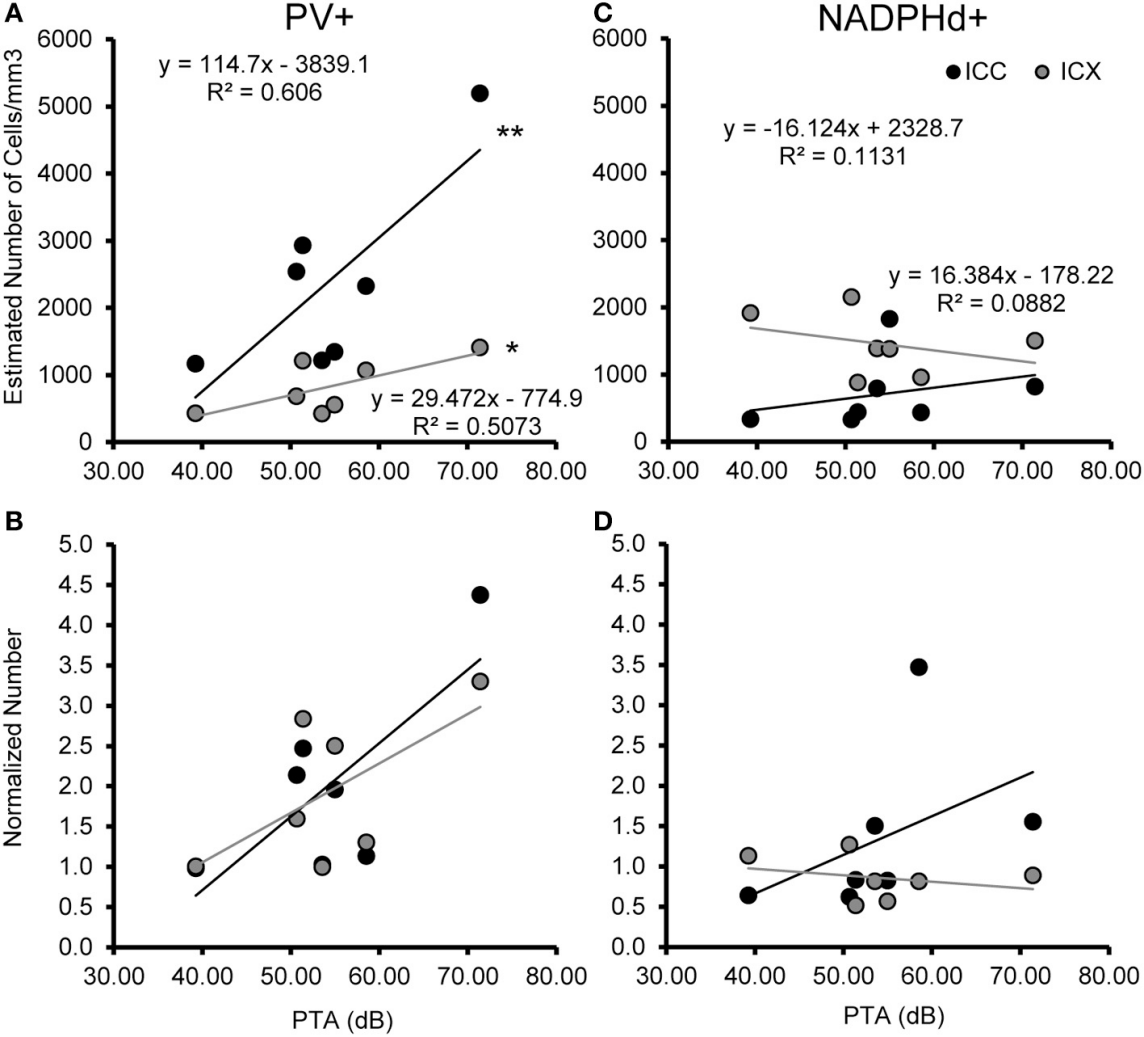
**Numbers of PV+ cells, but not NADPHd+ cells are correlated with ABR thresholds in the ICC and ICX**. The estimated number of PV+ **(A,B)** and NADPHd+ **(C,D)** cells in the core (black) and shell (gray) of the IC as a function of ABR PTA. The number of PV+ stained cells was significantly correlated with PTA for both ICC and ICX **(A)**, but this relationship did not hold for the number of NADPHd+ stained cells **(C)**. The number of PV+ **(B)** and NADPHd+ **(D)** stained cells normalized to the two youngest monkeys showed the same trends as seen for the correlation with age (compare Figures 6, 7). ^*^p < 0.05, ^**^p < 0.01.

We examined the relationship between age and ABR PTA thresholds and the estimated number of PV positive cells in the ICC and ICX of the IC. First, we found that the average estimated number of PV positive cells in the ICC (*r* = 0.94, *p* < 0.001) and the ICX (*r* = 0.93 *p* < 0.001) increased as a function of age (Figure 6). To explore this relationship further, we ratio normalized the number of the PV positive cells in the ICC and ICX of the two youngest adult monkeys and compared this value to the other five animals. This revealed that the number of PV positive cells gradually increased by 20 years of age and ended up being 4.3 times greater in the oldest monkey than what was observed in the young adult monkeys. Next, we examined whether ABR PTA was associated with the number of PV positive cells in the ICC and ICX of the IC. We found that the number of PV positive cells in the ICC (*r* = 0.85, *p* < 0.01) and the ICX (*r* = 0.69, *p* < 0.05) significantly increased as a function of increasing ABR PTA (Figure 7). To explore this relationship further, we ratio normalized the number of PV positive cells in the ICC and the ICX as a function of the ABR PTA. This revealed a similar result to the aging normalization except that the gradual increase in the number of PV positive cells was less predictive than the aging normalization.

The previous analysis revealed that the number of PV positive cells in the IC was associated with both the age of the monkey, and increases in hearing thresholds across the audiometric range. In order to determine if the increase in the number of PV positive cells in the IC is a compensatory mechanism to normal aging and hearing threshold, we used backwards multiple regression analysis to determine which explanatory variable best predicts the number of PV positive cells in the ICC and ICX of the IC. Using this strategy, we found that aging was the only significant predictor of the number of PV positive cells in the ICC of the IC [*b* = 0.944, *t*_(6)_ = 6.399, *p* < 0.001]. In our model, age accounted for 88% of the variance in the number of PV positive cells in the ICC of the IC [adj *R*^2^ = 0.87, *F*_(1, 6)_ = 40.953, *p* < 0.001]. We found a similar relationship in the ICX of the IC [*b* = 0.928, *t*_(6)_ = 5.562, *p* < 0.01] where age accounted for 83% of the variance in the number of PV positive cells [adj *R*^2^ = 0.833, *F*_(1, 6)_ = 30.934, *p* < 0.01].

### The number of NADPHD positive remains stable with age

We examined the relationship between age and ABR PTA to the estimated number of NADPHd positive cells in the ICC and ICX of the IC. Unlike the results that we found with PV, we found that the average estimated number of NADPHd positive cells in the ICC (*r* = 0.078, *p* > 0.05) was not associated with age (Figure 6). However, the estimated number of NADPHd positive cells in the ICX had a tendency to decrease with age (*r* = −0.321, *p* > 0.05). Normalizing for the number of the NADPHd positive cells to the youngest monkeys revealed that the number of NADPHd positive cells has a tendency to gradually decrease with age. Next, we examined whether ABR thresholds were correlated with the number of NADPHd positive cells in the ICC and ICX of the IC. We found that the number of NADPHd positive cells had a non-significant tendency to be related to the ABR threshold in the ICC (*r* = 0.315 *p* > 0.05), but not the ICX (*r* = −0.072, *p* > 0.05; Figure 7). Again, we normalized the number of NADPHd positive cells in the ICC and the ICX to the adult and plotted the relationship as a function of ABR threshold, revealing that the number of NADPHd positive cells is not related to ABR threshold within our sample.

The previous analysis revealed that the number of NADPHd positive cells in the IC was not significantly associated with either age or ABR threshold. To confirm these results, we used stepwise multiple regression analysis to determine if age or ABR threshold can be predicted by the number of NADPHd positive cells the ICC and ICX of the IC. This strategy confirmed the correlation results in that neither age nor ABR threshold reached the criteria to enter the model.

### The magnitude of the difference in the number of neurons between the ICC and ICX changes with age

**Figure 8:**
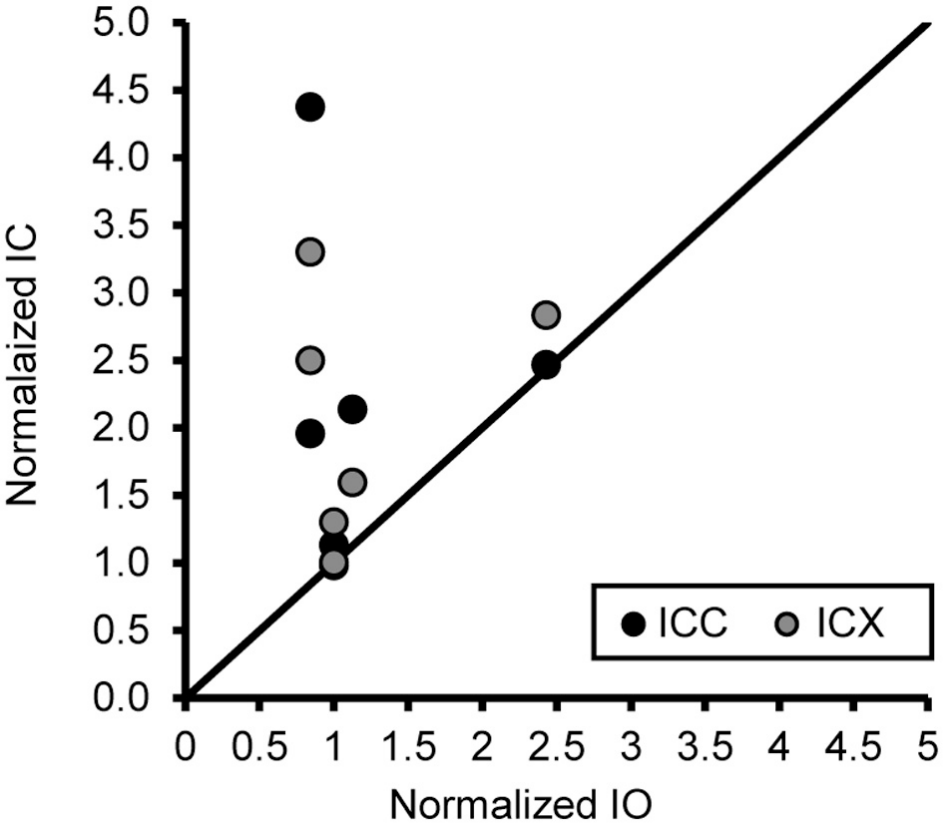
**An increase of the number of PV+ stained cells with age does not generalize across different nuclei in the same histological section**. The number of PV+ stained cells in the ICC and IO (black circles) and ICX and IO (gray circles) was normalized to that of the 15 year old monkey. The unity line represents no difference in the normalized number between in the IC and IO. There was no systematic relationship in the number of PV+ stained neurons between the inferior colliculus and inferior olive, indicating that the age-related changes in the ICC and ICX cannot be attributed to general anatomical changes or histological differences as a function of age. Note: Data included above are from cross sectioned tissue.

One concern is that the increase in PV positive neurons is not due to age or hearing thresholds, but rather to technical factors unrelated to aging. Examples would include differences in vascularization of the tissue, perfusion of the fixative, or differences in membrane properties that are non-specific to auditory regions. To address this issue and to examine the ratio of the number of PV positive neurons between the ICC and ICX, we conducted the same analysis of PV positive cells in the inferior olive nucleus (IO) of the cross-sectioned tissue, which was located in and adjacent to sections of the IC that was analyzed as described above. In this way we could make direct comparisons between two different neural structures that were located in the same tissue section and using identical methodology. We found that the number of PV positive cells in the IO was not significantly correlated with age (*r* = 0.14, *p* > 0.05) or changes in ABR threshold (*r* = −0.31, *p* > 0.05). Next, we examined the relationship between the number of PV positive cells between the IC and the IO. Similar to the previous results, we found that the number of PV positive cells did not systematically vary between the IO and the ICC (*r* = 0.06, *p* > 0.05) and ICX (*r* = 0.34, *p* > 0.05). Next, we ratio normalized the counts within the IO and IC to the counts found in the youngest monkey, and plotted the results against each other to illustrate the lack of correlation between these two areas (see Figure 8). This plot illustrates that the increase in the number of PV positive cells in the IC are not related to a systematic increase in the number of PV positive cells in the non-auditory area IO. Not surprisingly, similar results were found for NADPHd. These results strongly suggest that the relationships that we reported above are probably due to aging and changes in audiometric sensitivity rather than unrelated technical factors that may be present when working with aged tissue specimens.

## Discussion

This study was designed to enhance our understanding of the neurochemical correlates associated with age-related changes in the auditory system that has been reported in aged populations (O'Neill et al., 1997; Zettel et al., 1997; Ouda et al., 2003, 2008). Our results for the monkeys included in this study demonstrate that the ABR evoked pure tone average increases with age, although ABR to clicks remained relatively stable with age. In the inferior colliculus, the ICC and ICX were neurochemically distinct in their relative reactivity for PV and NADPHd, and represent the chemically defined parallel pathways that have been previously described in rodents, carnivores and monkeys (Jones, 2003; Loftus et al., 2008). We found that aging and changes in ABR thresholds across the audiometric range was accompanied by an increase in the number of PV positive cells in the ICC and the ICX of the IC. This, however, was not the case within our sample for the number of NADPHd positive cells in the ICC and ICX of the IC. These results suggest that one of the central effects of aging and changes in hearing sensitivity may be to up-regulate the expression of PV within some neurons in the IC.

### Auditory brainstem response to clicks and tones

The present finding regarding the shifts in ABR thresholds across the audiometric range is consistent with previous studies of rhesus macaque monkeys (Fowler et al., 2002, 2010; Torre et al., 2004; Engle and Recanzone, 2012). We found that the ABR wave IV threshold was within 10 dB of our middle-aged adult group with the exception of the oldest animal (35 years old), which had a 30 dB increase in threshold. In a recent study, Fowler et al. (2010) found a similar elevated pattern to clicks in their oldest animal (28 years old). We are aware that some of these monkeys may have experienced audiometric changes to tones at higher frequencies not directly examined here, and that neurochemical changes in the IC may be result of audiometric changes at the highest frequencies. Future investigations should explore how changes in processing specific frequencies relate to neurochemical changes in lower auditory structures along the auditory pathway from the cochlear nucleus to auditory cortex to elucidate this issue.

It is possible that changes in cochlear function were manifested outside the range of the click and tone stimuli (0.5–16 kHz) stimuli used here. Bennett et al. found an age-related increase of thresholds for high frequencies in their middle-aged animals, and elevated thresholds across the entire frequency range in their oldest rhesus monkeys (Bennett et al., 1983). Similar high frequency impairments have been found in marmosets (Harada et al., 1999). In a recent longitudinal study, Fowler et al. (2010) found that these threshold impairments increase at 21 years of age in monkeys. This high frequency impairment would be inherited by the IC, and could potentially influence the results in the high frequency region of the tonotopically organized ICC (Oliver, 2005). However, any compensatory change as the result of high frequency impairments should affect the results equally in the aged monkeys, and would only have negligible result on the number of PV and NADPHd cells in the IC. More comprehensive studies that directly relate changes in cochlear function and central anatomy are necessary to determine if distinct cochlear pathologies cause specific neurochemical compensatory signatures.

### Characterization of the IC of the macaque monkey

The IC of the macaque monkey was characterized by its relative reactivity for both PV and NADPHd. We found that the IC was organized into a PV dense ICC and a NADPHd rich ICX in a similar fashion to what has been recently reported in the mouse, rat and cat (Loftus et al., 2008). In primates, Parvizi and Damasio (2003) used the calcium binding protein calbindin in addition to PV to parcellate the ICC from the ICX. Interestingly, NADPHd has been found to colocalize in a region-specific manner with specific calcium binding proteins. For example, NADPHd co-localizes with calbindin, but not PV, in the cerebral cortex (Yan et al., 1996; Smiley et al., 2000). However, in the rat superior colliculus, NADPHd co-localizes with PV, but not with calbindin or calretinin (Soares-Mota et al., 2001). Although it was not directly tested in the present study, the distribution pattern of NADPHd and PV in the IC suggests that NADPHd may colocalize with the calcium binding proteins that are densely found in the ICX, such as calbindin and calretinin.

### Relationship to previous age-related studies

We were able to tease apart the effects of normal aging and hearing loss on the number of cells in the IC by characterizing the IC into two distinct neurochemically defined streams. We found that the number of PV positive cells increased with age in the IC of the macaque monkey, and that this increase was also related to changes in the ABR threshold across the audiometric range. Previous studies in rodents have found significant increases in the number of PV positive cells in the cochlear nucleus of the C57/BL6 and BALBc mice (Idrizbegovic et al., 2003, 2004, 2006) suggesting that there is a mechanism that compensates for peripheral pathologies that result in a reduction in drive from the cochlea. Additionally, similar changes have been found along the auditory pathway in the rhesus macaque, however, the observed compensatory changes appear to be regionally and chemically specific. For example, we have recently found that the number of NADPHd positive cells, but not PV positive cells, increase with age and ABR thresholds in the cochlear nucleus of macaques (Gray et al., 2014b). A different pattern appears at the next step along the auditory pathway in the superior olivary nucleus. Gray et al. (2013) found that the number of NADPHd and PV positive cells increased with both age and increases in ABR thresholds in the medial superior olivary nucleus of macaques. Here, we found that the number of PV positive cells, but not NADPHd cells, increased with age and changes in audiometric sensitivity. This trend continues in the medial geniculate, where there were more PV positive cells in the ventral division compared to the dorsal or magnocellular division with age (Gray et al., 2013, 2014b). No age-related studies of NADPHd positive cells have been conducted in the macaque auditory thalamus to our knowledge. These combined results suggest a compensatory change to simple acoustic processing throughout the ascending auditory pathway. It is, however, unclear if the increase of the cells types has a functional advantage on more complex auditory processing. It is possible that the increased number of PV positive cells in the IC of the aged monkeys is a mechanism to increase the efficacy of excitation or inhibition by up-regulating the number of PV positive cells to process simple acoustic features. Future studies in aged monkeys using complex stimuli are needed to determine if the age-related increase in the number of PV positive cells has a functional advantage on auditory processing.

Finally, given that there is an increase in the number of PV positive neurons in the aging ICC, the question is raised as to what this increase functionally means. PV is a calcium-binding protein that has been associated with fast-spiking inhibitory interneurons in the cerebral cortex and hippocampus (Kawaguchi et al., 1987; Cauli et al., 1997; Kawaguchi and Kubota, 1997; Gibson et al., 1999; Petilla Interneuron Nomenclature et al., 2008; Xu and Callaway, 2009). Co-localization studies in rodents (Fredich et al., 2009) have shown that in the central nucleus of the inferior colliculus, all GABAergic neurons also express PV. However, a significant majority of PV neurons were not GABAergic, but rather co-localized with glycine (10%) or with none of the amino-acid markers tested, implicating a neuromodulatory role (5%). GABAergic activity have been shown to decrease in the inferior colliculus with increased age in the rodent, but the response to GABA by the post-synaptic cell has been shown to increase (reviewed by Caspary et al., 2008). Thus, it is likely that the age-related increase in PV expressing cells is reflecting a compensation for an overall decrease in inhibitory drive in the inferior colliculus. How this increase in the number of PV positive neurons comes about is currently unclear. It is plausible that neurons convert from non-PV to PV expressing cells, that non-PV expressing cells are somehow selectively decreased, or that neurons that have levels of PV expression that are below that which the current techniques can visualize increase their expression to become clearly labeled. Co-localization studies with aged tissue will be necessary to resolve this question.

### Technical considerations

Our findings of age-related increases in PV positive neurons but not NADPHd positive neurons could be due in part to limitations of these neuroanatomical techniques. First, it is unlikely that our observers were biased in their ability to correctly identify positively labeled neurons given the relatively low background staining as well as the high level of agreement between observers and the accuracy relative to the thresholding control described in Gray et al. (2013). However, this does not preclude the possibility that the specific amounts of protein and enzyme expressed by the different cells did in fact change as a function of aging but were not detected by our methods. For example, low levels of constituent expression could have been increased but below the threshold of what would be characterized as positively labeled by our observers. More sensitive measures, such as Western blots, could be used to make this distinction in future experiments. Additionally, the stereological method that we applied to quantify these changes in our tissue may have introduced a systematic error in our estimates. Another shortcoming is that we counted neuronal cell bodies instead of the nucleus or nucleolus of each cell due to the nature of working with DAB stained tissue, which may have led to double counting of large cells across adjacent sections. Future studies can overcome these shortcomings by using design based stereological procedures on tissue that is double-labeled to confirm that the changes that were identified in this study are accurate.

A second consideration is that these data are based on seven monkeys, which is a relatively small sample given the span of ages tested. It was for this reason that we used regression analysis to better identify age-related trends as opposed to only grouping animals into different aged categories. Similarly small sample sizes, including a large overlap with the animals described in this study, have been used previously investigating other ascending auditory nuclei (Gray et al., 2013, 2014a,b). This small sample size is due in large part to the low availability of aged macaques for this class of studies, particularly those over 25 years of age. Indeed, the 35 year old monkey used in this study is the second oldest macaque reported for any auditory studies to our knowledge. Torre et al. (2004) reported one animal 36 years old, whereas Bennett et al., 1983 reported on a 31 year old. Fowler et al. (2002, 2010), and Torre and Fowler (2000) reported their oldest animals as 28 years or less. Future studies using a different and larger population of monkeys may indeed reveal significant changes where we only saw trends, which is certainly possible given our relative rigid criteria for statistical significance. The data report here can be easily incorporated into such future studies as the ages and cell counts are provided in the regression analysis plots (Figures 6, 7).

## Summary

In summary, the results of this study indicate that normal aging increases the number of PV positive cells in the IC. By using multiple regression analysis we teased apart the relative contribution of aging and hearing loss on the number of PV positive cells in the IC. We found that the number of PV positive cells increased as a function of age. It is possible that this age-related increase has a functional advantage on auditory processing, as observed in monkeys with increased PV positive densities with significant changes in the processing across the audiometric range. If this up-regulation is generalized throughout the auditory pathway, it predicts that processing simple acoustic features should be preserved in the auditory cortex. How this compensatory feature influences complex acoustic processing remains to be elucidated.

## Author contributions

Study concept design: James R. Engle and Gregg H. Recanzone. Acquisition of data: James R. Engle, Heather Turner, Daniel T. Gray, and Julia B. Udell. Analysis and interpretation: James R. Engle, Heather Turner, and Gregg H. Recanzone. Drafting the manuscript: James R. Engle and Gregg H. Recanzone. Critical revision of the manuscript: Daniel T. Gray. Statistical analysis: James R. Engle and Gregg H. Recanzone. Obtained funding: James R. Engle and Gregg H. Recanzone. Study supervision: Gregg H. Recanzone.

## Funding

Funded in part by NIA R21AG024372, R01AG034137 (Gregg H. Recanzone), and NIDCD T32DC008072 (James R. Engle).

### Conflict of interest statement

The authors declare that the research was conducted in the absence of any commercial or financial relationships that could be construed as a potential conflict of interest.

## Abbreviations

ARHL: Age-related hearing loss
ABR: Auditory brainstem response
DAB: 3,3′ diaminobenzidine
dB: Decibel
DC: Dorsal cortex of the inferior colliculus
LC: Lateral cortex of the inferior colliculus
IACUC: Institutional Animal Care and Use Committee
IC: Inferior Colliculus
ICC: Central nucleus of the inferior colliculus
IM: Intramuscular
IV: Intravenous
NADPHd: NADPH-diaphorase
PV: Parvalbumin
SPL: Sound pressure level.
